# A Study of Differences in Compulsory Courses Offering Medicine Humanization and Medical Communication in Polish Medical Schools: Content Analysis of Secondary Data

**DOI:** 10.3390/ijerph182413326

**Published:** 2021-12-17

**Authors:** Joanna Dec-Pietrowska, Agnieszka J. Szczepek

**Affiliations:** 1 Faculty of Medicine and Health Sciences, University of Zielona Góra, 65-046 Zielona Góra, Poland; 2 Department of Otorhinolaryngology, Head and Neck Surgery, Charité-Universitätsmedizin Berlin, Corporate Member of Freie Universität Berlin and Humboldt-Universität zu Berlin, 10117 Berlin, Germany

**Keywords:** medical humanization, medical communication, medical schools, curriculum

## Abstract

Medical humanity is an essential element of medical education, and the respective courses are introduced to the curricula of medical schools worldwide. However, significant differences in this type of medical education were identified in Italy, Spain, and the UK. In Poland, this issue was not yet analyzed. The classes offered on a compulsory and not elective basis secure the uniform skills of future physicians. Therefore, we were prompted to ask a question: do Polish medical students receive equal compulsory education in medical humanities? To answer that question, we performed a content analysis of mandatory classes’ frequency, types, and content on medical humanization and communication in Polish medical schools. The study used publicly available information provided on the home pages of the universities to perform content and comparative analyses. Of 22 identified universities, 15 had publicly listed teaching programs, and nine had freely available syllabi. The names and types of courses varied from school to school. The number of hours the courses offered throughout medical education ranged from 15 to 216. In some medical schools, the classes were scheduled during the early, pre-clinical part of the study, whereas in other schools they were offered each year. The content of the courses always covered the topics of physician–patient communication but rarely offered protocols, such as the Calgary Cambridge guide. We conclude that the medical humanities represented by medical humanization and communication courses are included in the publicly available compulsory curriculum of most Polish medical schools. However, to secure equal education of future Polish physicians, there is a need to unify the medical humanities program.

## 1. Introduction

Medical humanities embrace modeling the view on medicine via a humanistic approach (e.g., arts or history), enriching the medical team’s competence, and improving patient–physician relations [1]. The importance of medical humanities as feeding the healthcare and medicine humanization and the incorporation of respective courses in the curricula of medical schools have long been recognized, and the social and ethical aspects of medicine already started to be included in medical programs in North America in the 1970s [2]. In 2014, there were about 2600 medical schools globally [3], and the equivalence of their teaching program concerning main professional subjects was challenging to assess. Therefore, it is no surprise that the presence, content, duration, and format of courses concerning medical humanization and humanities vary between the medical schools. The benefit of teaching medical humanities has been demonstrated in research studying the effect of teaching visual arts [4,5] or languages [5] in medical schools. In addition, a need for further extension of curricula in some medical schools, e.g., by teaching bioethics [6], has been identified.

Medical communication is a non-technical skill necessary for all healthcare workers. Two dimensions of medical communication are of importance in the education of medical students: communication with patients and communication within a medical team (also termed interprofessional communication). The first skill is the prerequisite for patient’s satisfaction [7], influencing the therapy outcome [8]. The second skill impacts satisfaction and the safety of patients [9]. In addition, interprofessional communication influences the efficacy and timeliness of care and the well-being of healthcare workers [10]. Medical students who completed the medical and interprofessional communication courses gained competence and confidence when dealing with problems and conflicts in clinical settings [11], also in cases when the skills were acquired during an online class due to the COVID-19 pandemic [12]. Notably, there is a known difference in teaching the communication skills between the programs for physicians and nurses, which might also be a source of conflict and miscommunication [13].

Medical education has been offered in Poland since 1364. In that year, the Polish king Casimir the Great funded the university in Cracow (Studium Generale) with three faculties, one of them being the faculty of medicine. Of twenty-two medical schools registered in 2021, only two (in Cracow and Warsaw) operated before the second WW. Eight were established in the early post-war times (Bialystok, Gdansk, Katowice, Lodz, Lublin, Poznan, Szczecin, and Wroclaw). Twelve of the medical schools in Poland, which is more than 50%, were established relatively recently, ranging from the eighties (Bydgoszcz—1984), through the beginning of the century (Rzeszow—2005; Olsztyn—2007) up to the very new medical schools, which have only existed for a few years (see Supplementary Table S1), reflecting the turbulent Polish history. There are three possible structures of Polish medical schools: an independent medical university (presently 10), a faculty of medicine operating within the university (currently 6), and a medical college associated with the university (currently 6). Eighteen medical schools are public and do not charge tuition fees as the Polish government subsidizes them, and four are private and charge fees ranging from EUR 9,200 to 14,700 per year [14]. All medical schools offer a 6-year-long (12 semester) teaching program for future physicians ending with the MSc in Medicine. After completing a 13-month-long postgraduate internship, the students pass the State Medical Examination to obtain a license to practice medicine. In 2020, there were 84,428 medical students registered as actively studying medicine in Poland (source: https://stat.gov.pl/obszary-tematyczne/edukacja/edukacja/, last accessed on 12 December 2021). In 2017, the average number of physicians per 1000 population in Poland was 2.7, making it the lowest in Europe [15] and explaining the trend in establishing new medical schools.

The challenges of medical education in Poland include problems with designing and implementing comprehensive classes on professionalism and social competencies of future physicians [16]. Another problem among Polish medical students is a tendency to stigmatize patients, e.g., with psychiatric conditions [17], attributed to insufficient teaching of patient–physician communication. In addition, there are recent reports about the mistreatment of medical students by their academic teachers [18]. These problems could be addressed by well-designed and implemented education in medical communication and humanization for students and their teachers. In addition, since 2020, medical education worldwide has been affected by pandemics, and many courses have moved online. Polish medical students perceive this change as not overall negative but challenging [19]. Of special importance were the technical difficulties experienced at the beginning of the COVID-19 era.

In Europe, medical communication and other humanistic courses began to be offered by the medical schools in the late 1970s and early 1980s in the United Kingdom [20,21], followed by the Netherlands [22], Denmark [23], and relatively recently by other countries such as Germany [24,25,26] or Belgium [27]. Research performed in Poland also identified a need for courses focused on medicine humanization and medical communication during early medical education [28,29]. Some of the Polish medical schools implemented the necessary changes in the curriculum and assessed their effectiveness [30]. Still, little is known about how many medical schools in Poland offer compulsory courses on medical humanities, the content and duration of the classes, and when they are implemented during the education process.

The presence and content of medical humanities courses offered by various medical schools in Europe were already analyzed in Italy and Spain [31] as well as in the United Kingdom (in comparison to the USA and Canada) [32]. Both publications identified several compulsory and elective humanities courses offered by the analyzed medical schools and found major differences between their number per study period, content, and format. In addition, Howick et al. found a surprising negative correlation between the rank of the medical school and the number of courses offered, implying that renowned medical schools offer fewer courses on medical humanities than the low-ranking ones [32].

Medical students sometimes transfer to another university during their studies and need to catch up on all compulsory subjects not taken to fulfill the teaching curriculum. We have noticed that the mandatory courses dealing with medical humanities often need to be retaken in such cases. However, in some other cases, the students provide transcripts from their former universities confirming that they are taking many more classes than the present school requires. That observation has prompted us to formulate the following research question: *do Polish medical students receive equal compulsory education in medical humanities?* In addition, we were curious about the publicly accessible information offered to future medical undergraduates and general society. In the present work, we attempted to answer our research question by analyzing the content of publicly available data, as in Poland, the presence or content of courses dealing with medical humanities and medical communication has never been investigated. The two disciplines (humanities and communication) intercalate and, in medical practice, impact the patient’s care [33]. Therefore, to gain a current view of the situation, we analyzed Polish medical schools’ teaching programs and curricula concerning humanization, medical team’s competence, and patient–physician relations.

## 2. Materials and Methods

### 2.1. Search Methodology

We used a general search and analysis approach described by Howick et al. [32]. To identify all accredited medical schools in Poland, we used an online government registry of universities in Poland (https://polon.nauka.gov.pl/opi/aa/rejestry/szkolnictwo?execution=e1s1, accessed on 4 September 2021) and performed a search using selection criteria “active” and “medical university”. The investigation identified ten medical universities; nine offered primary medical education and one advanced medical education.

Next, using a search engine https://www.otouczelnie.pl/artykul/14110/Kierunki-studiow-medyczne (accessed on 5 September 2021), we found non-medical universities providing medical education and identified other 13 medical schools. Together, 22 Polish universities offering medical education were identified and included in the analysis.

**Figure 1:**
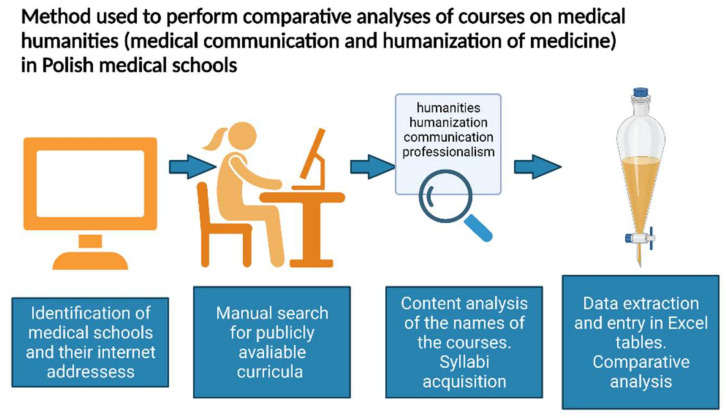
The methodological approach used in the study. Created with BioRender.com.

Using the internet addresses, we visited the home pages of each university and performed a manual search for the study plan and course description in the form of a syllabus (Figure 1). Of the initially included 22 medical schools, 15 provided public information on the study program, and 9 delivered publicly available syllabi.

### 2.2. Analyses

We performed a content analysis of the names of courses listed in the curricula by determining the presence of the following words: “humanization”, “humanities”, “communication”, “psychology”, “sociology”, and “professionalism”. Using this strategy, we identified several classes teaching humanization of medicine and patient–physician relations. In addition, we performed a manual search to identify courses that could offer related subjects but did not contain the selected keywords in their names. Next, we searched for and downloaded the syllabi of the respective classes and extracted the information on when (the year of study) and using which format (seminars, lectures, workshops) these courses are offered to the medical students who start their education in the academic year 2021/2022. In addition, we acquired information about how many teaching units (of 45 min) were allocated for each subject and which evaluation methods were used to assess the education effects.

We used a narrative synthesis to describe the observations made.

## 3. Results

### 3.1. Characteristics of Medical Schools Included in the Analysis

The 22 identified medical schools are based in all major cities of Poland. Cracow and Warsaw host more than one medical school (two and four, respectively). Ten medical schools have a rank of the autonomous medical university; six have the status of Collegium Medicum (medical college), and six are part of the Universities as Faculties of Medicine (see Supplementary Table S1).

### 3.2. Public Availability of Curricula and Syllabi

**Figure 2:**
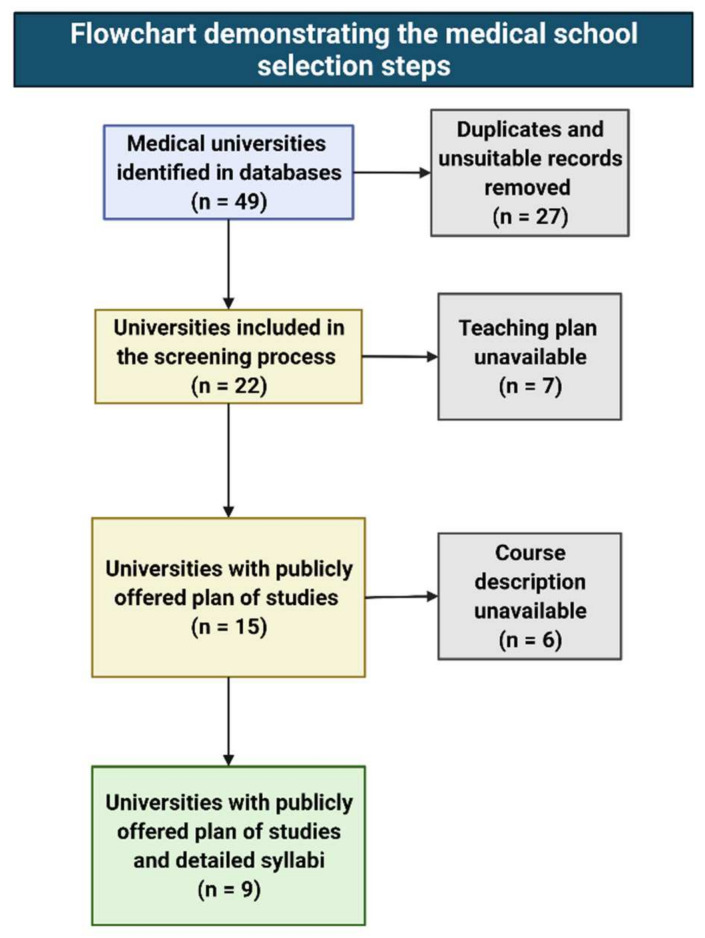
Selection steps during the analysis of course contents offered by the Polish medical schools.

Seven of the twenty-two identified medical schools had no publicly available teaching plans. The remaining 15 medical schools posted their curricula publicly with the names of the courses offered and information about the courses being compulsory or elective. Nine universities showed syllabi for the classes (Figure 2).

### 3.3. The Compulsory Courses Offered

Using the curricula, we identified compulsory courses and performed a content analysis of the descriptive names of the classes. For the study, we used the following keywords: “humanization”, “humanities”, “communication,” “psychology”, “sociology”, and “professionalism”. In addition, during manual search of syllabi we identified the following courses that were considered relevant and included them in comparative analyses: “Coping with stress”, “Generic competencies in medicine”, “How to be a physician”, “Integrated problem teaching” and “Practical teaching of clinical skills” The identified courses were offered under various names. The most commonly used in the course names were: “psychology” (seven courses) followed by “communication” (six courses), “professionalism” (six courses), and “stress” (two courses). The full names of the courses are listed in Table 1.

### 3.4. The Number of Courses Offered Per University, Level of Study, and Frequency of Teaching

**Figure 3:**
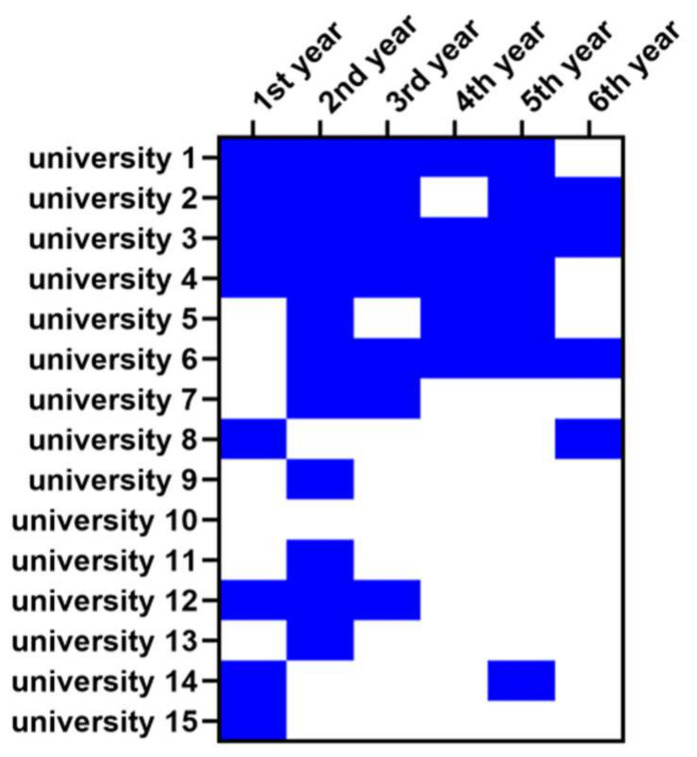
Heat map showing the frequencies with which the Polish medical schools teach the medical humanities courses. Blue color—a course offered; white color—course not offered. The universities are coded with numbers, and the coding is the same as in Table 1.

The number of compulsory courses on the humanization of medicine and communication varied from university to university. Based on the publicly accessible 6-year teaching program available for 15 medical schools, the number of compulsory courses offered throughout the medical study ranged from one to six. In addition, the frequency with which the courses were offered differed between the universities (Figure 3). The courses were taught by one university (#3) over the entire study period; two universities (#1 and #4) had planned the classes each year between the first and fifth year; one university (#6) between the second and sixth year of study. One university offered the courses between the first and third and between the fifth and sixth year (#2), and another university (#12) held the classes between the first and third year but not later. The rest of the medical schools planned the medical humanities for one or two years, predominantly at the beginning of the study.

Some universities offered classes under the same name (suggesting continuity of the teaching program) during each semester of study, whereas others implemented a class only once during the course of the medical program. Table 1 summarizes the information about specific classes. The names of the universities were replaced with random numbers.

**Table 1:** Short description of courses, including course name, the total number of teaching units per duration of the whole medical program (6 years), and evaluation methods. The information was extracted from the publicly available study program. The universities are coded with numbers the same as Figure 3 and Figure 4. OSCE—Objective Structured Clinical Examination.

University #	Course Name	Total Number of Hours	Evaluation Method
1	Generic competencies in medicine	216	credits with or without a grade
2	Professionalism	75	credits, OSCE exam
3	Medical communication; Medical psychology	68	credits
4	Psychosocial aspects of medicine	190	credit with grade
5	Professionalism in medicine	40	credit
6	Practical teaching of clinical skills	145	credit with a grade, OSCE
7	Medical communication; Sociology of medicine with elements of professionalism	54	No data
8	Integrated problem teaching; Medical psychology; Medical communication; Professionalism	No data	credit with grade
9	Humanization of medicine; Medical psychology	75	credit with grade
10	Elements of professionalism; Medical psychology; Coping with stress	55	credit with grade
11	Clinical communication	30	No data
12	Medical psychology	24	credit
13	Medical psychology; Doctor–patient communication	75	credit with grade
14	How to be a physician Advanced communication	15	pass (no grade)
15	Interpersonal communication; Medical psychology; Stress and human functioning	60	pass

### 3.5. The Format of Teaching and Evaluation Methods

**Figure 4:**
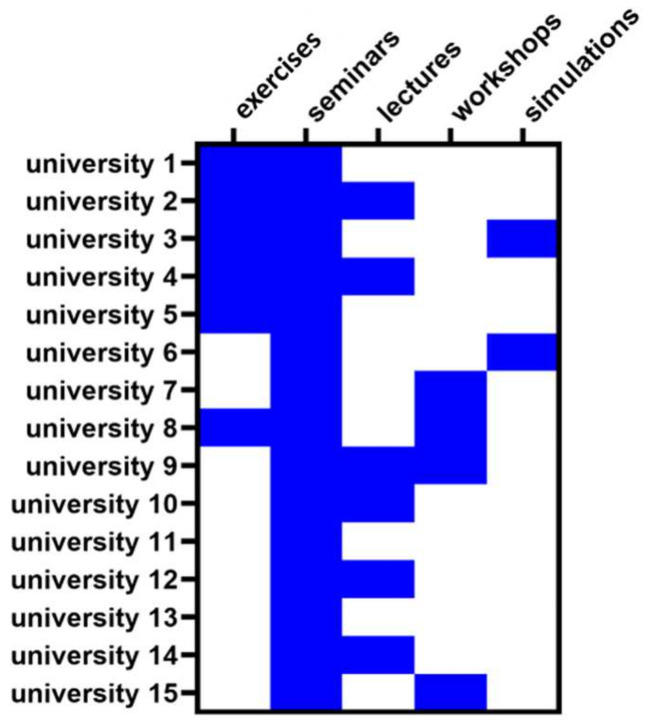
Heat map showing the frequencies with which the Polish medical schools use various formats to teach the medical humanities. Blue color—teaching format used; white color—teaching format not used. The universities are coded with numbers, and the coding is the same as in Table 1 and Figure 3.

The courses were offered in the following formats: exercises, seminars, lectures, workshops, and simulations. Seminars were the most popular form of teaching and were offered by all medical schools (Figure 4), followed by lectures and exercises (used by six medical schools), workshops (used by four medical schools), and simulations (used by two medical schools).

To evaluate the knowledge gained by students, the medical schools used various outcome measures (Table 2). Two medical schools used OSCE exams, nine schools used credit-based evaluation, two applied the “pass or no pass” system, and two provided no data.

### 3.6. The Content of Courses

In the next step, we analyzed the detailed description of all compulsory courses on the humanization of medicine and communication (Table 2). Such description was available from nine of twenty-two analyzed medical universities in the form of publicly available syllabi. The topics dealing, for instance, with the basics of communication between the physician and the patient and the patient’s family, and between medical team members, as well as verbal and non-verbal communication, were included in the compulsory courses offered by all universities. The majority of universities had a topic dealing with conflict situations included in their curricula. Some topics were included in the courses offered by four or five but not all universities (e.g., motivational conversation). However, there were also topics rarely included in the program of more than one or two universities (e.g., post-traumatic stress disorder, dealing with domestic violence, or Calgary–Cambridge guideline for patient–physician communication).

## 4. Discussion

The analyses of medical humanities courses were performed for a few European countries [31,32] but not Poland. The previous work determined differences between the curricula of various universities within the UK, Spain, and Italy regarding medical humanities. Likewise, our investigations based on the publicly available data posted by the universities on the Internet determined a great variety between the medical schools in Poland. The differences regarded whether the courses are offered and when, for how long, and what their content was. However, the previous publications focused on teaching anthropology, history, language studies, literature, music, philosophy, religion and theology, sociology, or visual arts, whereas we focused on teaching medical communication and the humanization of medicine as core medical competencies. Although the medical humanities started to be introduced to medical schools several decades ago [34,35], the differences in programs still exist today [32]. Analysis of teaching programs in medical humanities in medical schools based in the USA, Canada, and the United Kingdom determined a negative correlation between the quality of the medical school (as per international ranking) and the quantity and quality of the medical humanities courses offered [32]. We could not perform such a correlation because of the small sample size and a lack of ranking for 50% of the analyzed medical schools due to their very short existence. Nevertheless, performing such an analysis would be of value to the community in the future.

The need to introduce humanities-related classes to Polish medical schools was identified before [28,29], and many universities recognized it by including the respective courses into their curricula. However, several new medical universities have been established in Poland, whereas the older medical schools were restructured in the last ten years. We evaluated the compulsory courses concerning medical humanities, mainly dealing with physician–patient communication and competence in the medical team offered by the Polish medical universities to students who started their education in 2021/2022. The content analysis was based on publicly available information posted by the universities online and took into account the topics, frequency of teaching, and the content of courses. Below, we discuss the results of our research.

### 4.1. Public Availability of Data Posted by Medical Schools Requires Improvement

Our first observation concerns the public availability of teaching programs and syllabi, limited to fifteen and nine medical schools, respectively. Notably, the course content was available in some schools only upon logging into the system. In addition, the information available was often incomplete—something that was also noted for the USA-based medical schools over ten years ago [36]. This situation needs improvement to provide the prospective students with the possibility to obtain information about the detailed teaching plan before enrolling in the program. We suggest this improvement not only for Polish but worldwide medical schools.

### 4.2. The Number of Mandatory Classes, Level of Study, and Frequency of Teaching Differs between the Programs

We determined that medical schools offer various compulsory classes at different semesters of the study program. Some schools provided compulsory courses on medical competence and communication ongoing throughout the study (six of fifteen universities). However, some other schools settled down for teaching the subjects once or twice during the six years of the program. Furthermore, the total teaching volume dedicated to medical competence and communication differed significantly and ranged between 15 and 216 h of the compulsory courses offered. Moreover, the content of courses varied between the schools. All classes offered by nine medical schools with available syllabi contained components on the physician–patient relationship, communication with the patient, communication with the patient’s family, communication and cooperation with other medical team members, and verbal and non-verbal communication. However, most of the components (Table 2) varied between the universities, and some (e.g., learning about empathy, dealing with domestic violence) were offered only by one or a couple of schools. These results agree with the studies performed for Italy, Spain, the UK and Canada, and the USA [31,32], suggesting a global need for standardization of at least core subjects regarding the frequency of teaching and duration of compulsory classes on humanities and communication. Such equality was seen, for instance, in chiropractic teaching institutions’ gross anatomy classes regarding faculty, course design, delivery methods, and assessment methods [37].

The frequency with which the courses were offered throughout the studies, the duration and format of classes, and their content mirror various skills acquired in different medical schools and students’ preparedness for clinical reality. A study performed in Poland, in which the medical students and physicians have participated, identified a need for systematic education in professional communication skills during medical education [28]. Moreover, the same study stressed the need to evaluate skills appropriately. These skills should translate into improved professionalism (offered explicitly by six of nine analyzed medical schools), which, in Europe, was identified as a critical factor requiring improvement among physicians [38]. In addition, communication skills facilitating physicians’ contact with patients increase their satisfaction from medical care [39]. Although these skills can be acquired later in professional life, not only improving patients’ satisfaction but also decreasing burnout rate among physicians [39], learning medical communication early on enhances the performance of medical students already during their training [40].

### 4.3. The Format of Teaching and Evaluation Methods Vary between the Medical Schools

The most common format used by the schools was a seminar. In addition to seminars, six schools used lectures, six used workshops, and six used exercises. Despite proven value [41], the least-used teaching format was a simulation involving simulation patients. Various approaches were developed to teach communication in healthcare, and many of them (e.g., role play, group work, using simulation patients) were highly rated by medical students [42]. Only a few of these approaches were listed in syllabi that we analyzed, which does not necessarily mean that they have not been used. Here, improving structure and clarity of course description is recommended. Lastly, students’ knowledge evaluation fluctuated between “pass” to “OSCE exams”, which could also contribute to the ultimate knowledge of the medical students. Studies performed by one medical school in Poland determined the benefits of OSCE examinations and the student’s satisfaction from this type of evaluation [43], strongly suggesting a need for change in that particular area of medical education for the whole country.

### 4.4. Remote Teaching

Notably, none of the programs or syllabi have specified the possibility of using remote teaching, although one can assume that this format has undoubtedly been used since the beginning of the COVID-19 pandemic (spring semester 2020—onwards). The pandemic created a challenge in medical education worldwide and revealed compromised preparedness of medical students and teaching institutions for digital remote learning also seen in Poland [19]. However, the overall remote teaching/learning experience was rated as favorable by 73% of Polish medical students [44]. Importantly, the prospective medical students rated the remote classes on medical humanities positively, and the evaluation of their skills acquired during the course determined no significant difference compared to pre-pandemic times [45]. It is tempting to speculate that embracing online learning (and teaching) as an alternative or additional study format could improve education in the future. Remote participation of students and faculty, who, for some reason, cannot attend stationary courses (e.g., parental leave, illness restricting only the mobility of an individual, or employment of foreign faculty members) would decrease the rate of called-off classes and the rate of absence. It could also enrich the curricula, for instance, by introducing online counseling or telemedicine for physicians—a skill needed not only during pandemics [46] but also in rural or difficult to reach areas or to access patients with restricted mobility [47]. However, further research should determine which particular humanization and communication skills (including interdisciplinary communication) should be focused on. We suggest an interdisciplinary and international effort to address the issue to avoid creating further education gaps between the medical schools, areas, and countries.

### 4.5. Limitations

Our work is not free of limitations. Only two authors searched, screened, and analyzed data. Although we performed a crisscross check for the accuracy of search and analyses, this still creates a greater possibility for human error than when involving a more significant number of people. We plan to engage undergraduate medical students in similar research in the future. The second limitation is our choice of keywords, which could have led to missing some of the courses. We tried to complement this pitfall by performing a manual search outside of the keyword scope, but the possibility of omitting a course teaching communication and humanization still exists. We suggest introducing the keywords into course descriptions, as this could be a step in unifying and equalizing teaching programs in medical humanities. The third limitation of our study is that we concentrated on curricula offered for the medical students who start their education in the academic year 2021/2022. Because of the pandemic, not all administrative duties were fulfilled 100% by all universities, possibly resulting in incomplete information posted on the web. It is tempting to speculate that some of the missing data could have been made public by now, affecting the results and conclusion of our research. Another limitation is that we analyzed only the compulsory and not elective classes. We came across many interesting elective courses in medical humanities during our search. However, all elective courses had a limited number of attendees, and the present work aimed to compare the skills acquired by all medical students graduating from a medical school. The last limitation is the information about the teaching curricula. Because of the criteria set at the beginning of the study (public availability of data), we had to exclude the universities without freely obtainable curricula. Therefore, the excluded universities may still offer a rich program in humanities that was not considered in the present analyses.

### 4.6. Outlook and Future Directions

Despite the pitfalls of our work, our study delivers a broad picture of the variation in Polish medical schools regarding the mandatory teaching of medical communication and the humanization of medicine. As much as electives enrich the general medical education and are a welcomed but not a compulsory enhancement of curricula, the mandatory subjects define the comprehensive profile of all graduates. That is why we believe that the results of our work can be generalized not only for the Polish but worldwide-based medical schools, calling for the unification of medical teaching regarding medical communication and the humanization of medicine.

Improper communication in healthcare has multiple consequences, starting from negative patient experiences and ending in fatal medical errors (reviewed in [48]). Moreover, healthcare humanization was identified as essential not only for the patients but also for the healthcare professionals [49]. Thus, offering the basics of both subjects to medical undergraduates in the form of compulsory classes seems logical to provide future equal healthcare standards and warrant stakeholders’ (patients, patients’ caregivers, healthcare providers) satisfaction. Our research determined a positive trend in teaching medical communication and humanization and identified room for improvement, which could be addressed by equalizing teaching volume, content, and methods.

Future research should address the patient perspective on changes in medical education regarding medical humanization and communication. Such a study could identify the areas that could be emphasized in the program. Moreover, international comparative research on that topic could deliver country-, area-, or language-specific issues that need to be taken into consideration by medical students who study abroad.

## 5. Conclusions

Many medical schools embraced the medical humanities curricula concerning patient and medical team communication in Poland. However, the compulsory courses offered by the medical schools differ regarding the duration of teaching, format, content, and forms of evaluation. These differences create a gap in medical education between the graduates from various schools and, in the long run, might be responsible for lower patient satisfaction and a greater rate of burnout among physicians. Finally, this work identified a need for homogenous education in medical humanities and communication at Polish medical universities.

## Figures and Tables

**Table 2:** Analysis of the content of the compulsory courses offered. The information was extracted from nine universities that posted their syllabi online for public use. The universities are coded with numbers, and the coding is the same as in Table 1 and Figure 3 and Figure 4.

Topics	2	3	4	5	6	8	9	10	15
Physician–patient relationship	+	+	+	+	+	+	+	+	+
Communication with patient	+	+	+	+	+	+	+	+	+
Communication with patient’s family	+	+	+	+	+	+	+	+	+
Communication and cooperation with other members of the medical team	+	+	+	+	+	+	+	+	+
Verbal and non-verbal communication	+	+	+	+	+	+	+	+	+
Professionalism in medicine, the medical profession	+	-	+	+	+	+	-	+	-
Methods of constructive conflict resolution, solving problems and conflicts in the medical team	+	+	+	−	−	+	+	+	+
Clinical consultation and clinical interview	+	-	+	+	−	+	+	+	+
Communicating bad or unexpected news, talking about death	+	+	+	+	−	+	+	+	+
Difficult patients and difficult clinical situations	+	+	+	+	−	+	+	+	+
Focusing on patient’s needs	-	+	+	+	+	+	+	+	−
Managing the interaction with the patient, his family and cooperating medical staff	−	+	+	+	+	+	+	+	−
Physician’s role in in establishing and maintaining contact with the patient	+	+	−	+	+	+	+	+	+
Empathy	−	+	+	−	+	+	+	+	−
First contact with patient	+	−	−	−	−	+	+	+	−
Cultural, ethnic, linguistic, and religious aspects of communication with patients and their families	+	−	+	−	+	+	+	−	+
Motivating interview	−	+	+	−	+	−	+	−	−
Burnout, prevention, and treatment of burnout	−	+	+	−	+	+	+	+	+
Building trust in interaction with the patient	−	−	+	−	−	+	+	+	−
Coping with stress	−	+	+	−	−	+	+	+	+
Ways of dealing with emotions, anxiety, and aggression of the patient	−	+	+	−	−	+	+	+	+
Ethics of the medical profession	+	−	+	−	+	+	+	+	−
Patient’s rights	+	−	+	−	+	+	+	+	−
Communication barriers, errors and traps, language, jargon,	−	−	+	−	−	+	+	+	+
communication styles	−	+	+	−	−	+	+	+	-
Uncertainty communication	−	−	−	−	−	+	−	−	+
Medical errors	−	−	+	−	+	+	−	−	−
Motivating the patient to change	−	−	+	−	−	+	+	+	+
Patient’s perception and expectations	−	−	−	−	−	+	+	−	−
Interpersonal communication	−	+	+	−	−	−	+	+	+
The art of asking questions	−	+	−	−	−	−	−	+	+
Gender differences in communication	−	−	−	−	−	−	+	−	+
Assertiveness	−	+	+	−	−	−	+	−	+
Making recommendations	−	−	−	−	−	−	−	+	+
Stress, mental crisis	−	+	+	−	−	−	−	+	−
Relaxation training and stress-management techniques (mindfulness, autogenic training, progressive muscle relaxation, visualization)	−	−	+	−	−	−	−	+	−
Narrative medicine	−	−	+	−	−	−	+	−	−
Providing feedback in communication	−	+	+	−	−	−	+	+	−
Calgary–Cambridge guidelines	−	−	+	−	−	−	−	−	−
Dealing with post-traumatic stress disorder	−	−	+	−	−	−	+	−	−
Dealing with domestic violence	−	−	+	−	−	−	+	−	−
Empathy, ABCDE protocol	−	−	+	−	−	−	+	−	−
Ways to engage the patient in communication and to maintain contact with a patient	−	−	−	−	−	−	−	+	−

## Data Availability

Data are available upon request.

## Supplementary Materials

The following are available online at https://www.mdpi.com/article/10.3390/ijerph182413326/s1, Table S1: The Universities and Medical Schools identified in Poland are presented alphabetically based on the city’s name.
