# Supplementary material for: A Study of Differences in Compulsory Courses Offering Medicine Humanization and Medical Communication in Polish Medical Schools: Content Analysis of Secondary Data
Source: Int J Environ Res Public Health. 2021 Dec 17;18(24):13326. doi: 10.3390/ijerph182413326 (PMC8706785; doi:10.3390/ijerph182413326)
Supplement: Supplementary file 1 [file ijerph-18-13326-s001.zip › ijerph-1482479-supplementary.pdf]

Supplementary Table S1. The Universities and Medical Schools identified in Poland are presented alphabetically based on the city's name.

Name of the university	Web address	Year of establishment	City
Medical University of Bialystok	https://www.umb.edu.pl/	Established in 2008, as a result of the transformation of the Medical Academy in Białystok, operating since 1950.	Białystok
Collegium Medicum of the Nicolaus Copernicus University	https://www.cm.umk.pl/	In 1984, the Medical Academy was established in Bydgoszcz as a branch of the Medical Faculty in Gdańsk. On November 24, 2004, the Medical University of Ludwik Rydygier in Bydgoszcz was incorporated into the Nicolaus Copernicus University in Toruń as Ludwik Rydygier Collegium Medicum in Bydgoszcz.	Bydgoszcz
Collegium Medicum of the Jagiellonian University	https://www.cm-uj.krakow.pl/	The Faculty of Medicine was established in 1364 with the founding of the Cracow Academy. In 1950, it was separated from the Jagiellonian University under the name Medical Academy Nicolaus Copernicus in Krakow. The Academy returned to the Alma Mater in 1993 under the name Collegium Medicum of the Jagiellonian University.	Cracow
The Faculty of Medicine of the Krakow Academy Andrzej Frycz Modrzewski	https://www.ka.edu.pl/lek/	Created in 2016; first recruitment of medical students in 2016.	Cracow
Medical University of Gdańsk	https://gumed.edu.pl/	Established in 2009, as a result of the transformation of the Medical Academy in Gdańsk, operating since 1950. In the years 1945–1950, the Medical Academy operated in the city of Gdansk, earlier known as the Medizinische Akademie in Danzig and the Academy of Practical Medicine	Gdansk
Medical University of Silesia in Katowice	https://sum.edu.pl/	It was established in 2007 due to the transformation of the Silesian Medical Academy. Ludwik	Katowice

		Waryński, founded in 1948 as the Medical Academy in Bytom.	
The University of Technology in Katowice - Faculty of Medical Sciences	https://www.wst.com.pl/oferta_edukacyjna/kierunek_lekarski	The medical studies are offered from the academic year 2018/2019	Katowice
Collegium Medicum of Jan Kochanowski University in Kielce	https://cm.ujk.edu.pl/wydzial/o-wydział-cm/	Established in 2019 based on the Faculty of Medicine and Health Sciences of the Jagiellonian University, which has been educating in the field of medicine since 2015; 2015 Department Medicine and Health Sciences, from 2019 CM	Kielce
Medical University of Lodz	https://umed.pl/	Established in 2002 due to a merger between the Medical Academy (since 1950) and the Military Medical Academy (since 1958).	Łódź
Medical University of Lublin	http://www.umlub.pl/	Established in 2008, due to the transformation of the Medical Academy in Lublin operating since 1950. Bearing the name of Feliks Skubiszewski since 2002	Lublin
Collegium Medicum of the University of Warmia and Mazury	http://www.uwm.edu.pl/tag/collegium-medicum	Established in 2007, as a Faculty of Medical Sciences in Olsztyn, which in 2017 was transformed into the Collegium Medicum of the University of Warmia and Mazury; 2007 Department Nauk Med., from 2017 CM	Olsztyn
Faculty of Medicine, University of Opole	http://im.wmnoz.uni.opole.pl/ and http://im.wmnoz.uni.opole.pl/sylabusy-2019-2020/	entitled to teach medicine from the academic year 2017/2018	Opole
Medical University of Karol Marcinkowski in Poznań	http://www.ump.edu.pl/	Established in 2007, due to the transformation of the Medical Academy operating since 1950, which in 1984 was named after Karol Marcinkowski.	Poznan
Faculty of Medical Sciences and Health Sciences at the University of Technology and Humanities in Radom	https://wnminoz.uni Universityetradom.pl/	Licensed to teach medicine from the academic year 2017/2018	Radom
Medical Faculty of the University of Rzeszów	https://www.ur.edu.pl/kolegia/kolegium-nauk-medycznych/kolegium	Established in 2005 as a Faculty of Health Sciences. The name of	Rzeszow

		the Faculty of Medicine was adopted in the same year.	
Pomeranian Medical University in Szczecin	https://www.pum.edu.pl/	Established in 2010, due to the transformation of Pomeranian Medical University in Szczecin, created in 1948 as the Medical Academy in Szczecin and bearing the name of the Pomeranian Medical Academy in the years 1949–1992. General Karol Świerczewski	Szczecin
Warsaw Medical University	https://www.wum.edu.pl/	Established in 2008, as a result of the transformation of the Medical Academy in Warsaw, operating since 1950. Its roots go back to the Medical Academy, founded in 1809 in Warsaw	Warsaw
Medical Faculty. Collegium Medicum of the Cardinal Stefan Wyszyński University in Warsaw	https://wmcm.uksw.edu.pl/	Created in 2018. Recruiting medical students from 2019	Warsaw
The Faculty of Medicine of the Lazarski University in Warsaw	https://www.lazarski.pl/pl/wydzialy-i-jednostki/wydzial-medyczny/	Entitled to teach medicine from the academic year 2017/2018	Warsaw
Medical University of Maria Skłodowskiej-Curie in Warsaw	https://uczelniamedyczna.com.pl/	Offering medical education from the academic year 2020/2021	Warsaw
Medical University of Silesian Piasts in Wrocław	https://www.umw.edu.pl/	Established in 2012, due to the transformation of the Medical Academy in Wrocław. Operating since 1950; bearing the name of the Silesian Piasts since 1989. The Medical Academy functioned based on the Faculty of Medicine of the University of Wrocław, existed since 1945	Wroclaw
Collegium Medicum of the University of Zielona Góra	https://www.cm.uz.zgora.pl/	The Faculty of Medicine and Health Sciences was established in 2015 at the University of Zielona Góra; in 2019 transformed into the Collegium Medicum	Zielona Góra
